# Intrinsic Multienzyme-like Activities of the Nanoparticles of Mn and Fe Cyano-Bridged Assemblies

**DOI:** 10.3390/nano12122095

**Published:** 2022-06-17

**Authors:** Yunong Zhang, David Kudriashov, Liubov Pershina, Andreas Offenhäusser, Yulia Mourzina

**Affiliations:** Institute of Biological Information Processing (IBI-3-Bioelectronics), Forschungszentrum Jülich, 52425 Jülich, Germany; yun.zhang@fz-juelich.de (Y.Z.); d.kudriashov@fz-juelich.de (D.K.); liubovvldpershina@gmail.com (L.P.); a.offenhaeusser@fz-juelich.de (A.O.)

**Keywords:** nanozymes, electrocatalysis, manganese, Prussian blue, nanoparticles, hydrogen peroxide, reactive oxygen species

## Abstract

This study investigates the intrinsic multienzyme-like properties of the non-stabilized nanocrystalline nanoparticles of manganese-doped Prussian blue (Mn-PB) nanozymes and Prussian blue (PB) nanozymes in chemical and electrocatalytic transformations of reactive oxygen species. The effect of manganese doping on the structural, biomimetic, and electrocatalytic properties of cyano-bridged assemblies is also discussed.

## 1. Introduction

Transition metal complexes (TMCs) in macromolecular environments are used effectively in nature to perform a variety of functions, such as light harvesting, energy transformation, chemical modifications of molecules, and molecular binding and transport. TMC nanozymes are nanomaterials with enzyme-like properties based on transition metals. They have attracted increasing attention in recent years due to their biomimetic catalytic activities. TMC nanozymes can mediate (electro)chemical transformations using the ability of transition metals to accept multiple oxidation states and to provide coordination as well as molecular binding positions. Moreover, they are highly desirable for practical use, as they offer convenient long-term storage, the possibility of large-scale production, adjustable composition and properties, and effective direct electron transfer between redox centers and electrode surfaces, which is not hindered by enzymes’ insulating protein shells.

The search for efficient nanozymes that functionally mimic the major antioxidant enzymes-catalase, superoxide dismutase, and peroxidase represents a possible approach to control and use reactions mediated by reactive oxygen species (ROS). Since researchers had established a peroxidase mimetic Fe_2_O_3_ nanozyme in optical assay [1,2], investigations of transition metal oxides [3,4,5,6,7,8,9,10,11], macroheterocycles [12,13,14,15,16,17,18], and metal organic frameworks [19] have led to the discovery of further antioxidant (multi)enzyme mimetic compounds and electrocatalysts in chemical and electrochemical reactions mediated by ROS. One of these antioxidant enzyme mimetic compounds is Prussian blue (PB), which is referred to as an “artificial peroxidase” [20,21]. PB nanocolloids and a number of other cyano-bridged transition metal assemblies have a remarkably broad spectrum of applications, ranging from staining, sensors, and immunoassays [22,23,24,25] to energy storage [26], cesium and thallium adsorbents, and electrochromic devices.

Recently, peroxidase and catalase activity, as well as the superoxide scavenging ability of the PB nanoparticles (NPs) stabilized by various surface capping agents in chemical reactions, have been investigated with regard to the control of the cellular level of ROS [27,28,29]. The stabilized PB NPs have been proposed for applications as ROS scavengers in pathological processes, theranostics, and other biomedicine fields [27,30]. On the other hand, an investigation of the PB degradation process during H_2_O_2_ reduction by Nöel et al. [31] showed the formation of iron hydroxides and ferrocyanide ions in solutions along with the formation of the hydroxyl radical. Moreover, the cytotoxic effect of iron oxide NPs based on the generation of free radicals was shown [10]. Further studies led to a discussion about the dual function of PB nanoparticles as both scavengers and generators of free radicals and about their potential cytotoxicity [32].

However, it should be emphasized that stabilizing agents bearing various functional groups can, “beyond acting as a stabilizer” [32], change the acid-base and redox properties of nanomaterials and contribute to the apparent (electro)catalytic activity, with the result that the enzyme-like properties of the nanomaterials can be affected by the stabilizing agent. Moreover, similar to an insulating macromolecular shell, the stabilizing agent often hinders or even blocks electron transfer at the nanomaterial/electrode interface in electrochemical systems [33,34].

It is expected that the introduction of other transition metals into the PB structure may alter the multienzymatic activity of nanomaterials and promote the development of biomimetic arrays. Interest in investigating the catalytic properties of manganese complexes is due to their essential role in redox biology with regard to the composition of antioxidant enzymes: manganese-containing catalases, mitochondrial manganese superoxide dismutase (MnSOD), and manganese peroxidase, as well as a variety of Mn oxidation states that are favorable for (electro)catalytic activities. Moreover, Mn complexes are known for their high catalytic activity and protective role in (electro)chemical reactions with dioxygen and ROS [14,35,36,37,38,39,40]. Recently, Mn-doped PB nanoparticles were used in a biological environment as efficient agents in photothermal therapy [41] and MR imaging [42]. Previous studies have shown that key data on the intrinsic multienzyme properties and mechanisms of these nanomaterials, which are important for their practical use, are still not fully understood. In this study, we report on and compare the intrinsic multienzyme mimetic properties of non-stabilized nanocrystalline nanoparticles of manganese-doped Prussian blue (Mn-PB NCPs) and Prussian blue (PB NCPs) in chemical and electrochemical transformations of reactive oxygen species. The effect of manganese doping on the structural, biomimetic, and electrocatalytic properties of cyano-bridged assemblies will also be presented and discussed.

## 2. Materials and Methods

### 2.1. Chemicals

Bovine liver catalase (H_2_O_2_:H_2_O_2_ oxidoreductase EC 1.11.1.6, 2000–5000 units mg^−1^), xanthine oxidase (XO) from bovine milk (xanthine:oxygen oxidoreductase, EC 1.17.3.2, ≥0.4 units mg^−1^), peroxidase from horseradish (type VI, 310 units mg^−1^) (HRP), cytochrome *c* from equine heart (λ_max_^reduced form^ = 550 nm), 2,2′-azino-bis(3-ethylbenzothiazoline-6-sulfonic acid (ABTS), superoxide dismutase (1854 units mg^−1^) (SOD), acetone, 2-isopropanol, manganese(II) chloride tetrahydrate, and other analytical grade chemicals were purchased from Merck KGaA, Darmstadt, Deutschland. Phosphate buffers were prepared from sodium dihydrogen phosphate and disodium hydrogen phosphate. pH 2.0 and pH 3.0 buffers were prepared from phosphoric acid and sodium dihydrogen phosphate. The pH of the solutions was measured using a pH meter (Knick GmbH, Berlin, Germany). The O_2_ concentration was monitored using an oxygen sensor (LE621-IP67, MettlerToledo GmbH, Gießen, Germany).

All solutions were prepared using a Milli-Q water purification system (Merk KGaA, Darmstadt, Germany). Before the experiments began, the value of the extinction coefficient, ε_340_, of the ABTS product was examined, and it corresponded to 3.7 × 10^4^ M^−1^ cm^−1^ [43].

### 2.2. Synthesis of Nanoparticles

The nanoparticles were synthesized using chemical reduction and precipitation methods.

#### 2.2.1. Synthesis of PB NCPs

In this method, a mixture of Fe^3+^ and [Fe(CN)_6_]^3−^ with a high oxidation power (+0.98 V vs. Ag/AgCl) was used in combination with H_2_O_2_ as a reducing agent [21,44]. The use of H_2_O_2_ as a reducing agent allows artifacts to be avoided in the redox processes of the nanocrystals, which may appear as a result of the adsorption of redox active fragments of organic reducing agents and stabilizers on the surface of the produced nanocrystals. PB NCPs were synthesized by dissolving FeCl_3_ and K_3_[Fe(CN)_6_] at a 1:1 molar ratio in a supporting solution that contained 0.1 M HCl and 0.1 M KCl. PB nanoparticles were produced immediately after the addition of 30 wt% H_2_O_2_ as a reductant under ultrasonication. After 1 min ultrasonication, the reaction continued for another 5 min, and the resulting mixture was then centrifuged at 16,400 rpm for isolation. Subsequently, the nanoparticles were collected and dispersed into water for washing. This dispersion–centrifugation process was repeated seven times. Finally, the obtained nanocrystals were dried at 60 °C for 1 h and stored at room temperature.

#### 2.2.2. Synthesis of Mn-PB NCPs

Mn-PB NCPs were prepared by modifying the self-assembly reaction [41,42] of molecular precursors MnCl_2_, FeCl_3_, and K_4_[Fe(CN)_6_] to produce cyano-bridged bimetallic NCPs. Two solutions with a salt concentration of 0.05 M in 0.1 M HCl were prepared. Solution (1) contained iron(III) chloride and manganese(II) chloride, while solution (2) contained potassium hexacyanoferrate(II). Solution (1) was added dropwise to solution (2) under stirring. The resulting mixture was then stirred for 30 min. Subsequently, the product was centrifuged for 5 min at 6000 rpm, and the precipitate was washed and centrifuged for 5 min at the same rotation speed. Next, the precipitate was washed with deionized water and centrifuged for 5 min at 16,400 rpm. This step was repeated five times. The precipitate obtained after washing was collected and left to dry overnight.

### 2.3. Characterization Methods

#### 2.3.1. Scanning Electron Microscopy (SEM)

SEM and energy-dispersive X-ray spectroscopy (EDX) were carried out using a Magellan XHR SEM system equipped with an EDX detector (FEI, Hillsboro, OR, USA). The Mn-PB and PB NCP samples were deposited on Si/SiO_2_ or carbon substrates after being dispersed in water, dried, washed with deionized water, and finally dried again.

#### 2.3.2. XRD, XPS, and ICP-OES Analysis

X-ray diffraction (XRD) analysis was performed on a STOE θ/θ− diffractometer (STOE&Cie GmbH, Darmstadt, Germany) in reflection mode using Cu^−^, Kα, λ = 1.5418 Å radiation.

The X-ray photoelectron spectroscopy (XPS) investigation was carried out on a Phi5000 VersaProbe II (ULVAC-Phi Inc., Kanagawa, Japan).

Inductively coupled plasma optical emission spectroscopy (ICP-OES) was performed on a Thermo Scientific iCAP7600 spectrometer (Thermo Fisher Scientific, Waltham, MA, USA). 

#### 2.3.3. UV-Vis Spectroscopy

Ultraviolet-visible (UV-Vis) spectrophotometric measurements were carried out with a PerkinElmer Lambda 900 spectrophotometer (PerkinElmer, Waltham, MA, USA) using quartz cuvettes.

#### 2.3.4. Electrochemical Characterization

Electrochemical experiments were performed in a three-electrode cell controlled by an Autolab PGSTAT (Metrohm Autolab B.V., Utrecht, The Netherlands) and Nova software (Version 2.1, Metrohm Autolab B.V., Utrecht, The Netherlands). An Ag/AgCl double-junction reference electrode (Metrohm, Herisau, Switzerland) was used. A coiled platinum wire served as an auxiliary electrode. The supporting electrolytes were 0.05 M pH 3 and pH 7.4 phosphate buffer solutions with the addition of KCl to a concentration of 0.1 M.

### 2.4. Spectrophotometric Assay

#### 2.4.1. Peroxidase Activity Assay

A 1000 μL pH 2.0 buffer, 150 μL 30 wt% H_2_O_2_, 50 μL 10 mg mL^−1^ colorimetric substrate in buffer, and 50 μL solutions containing different concentrations of Mn-PB or PB NCPs in buffer were added to the 0.5 cm quartz cuvettes to perform the measurements. For the reference cuvette, 150 μL 30 wt% H_2_O_2_ was replaced by 150 μL DI water. All solutions were incubated at 37 °C in a water bath, with the exception of the 30 wt% H_2_O_2_ solution and DI water. In our experiment, 2,2’-azino-bis(3-ethylbenzothiazoline-6-sulfonic acid (ABTS, *λ*_max_ = 414 nm) was used as a colorimetric substrate. The reaction between ABTS and H_2_O_2_ was recorded by detecting the absorption intensity at 420 nm on a UV-Vis spectrophotometer. By using different concentrations of Mn-PB or PB NCPs in the reaction mixture, a series of *A*-*t* curves was obtained. The peroxidase-like activity was calculated using the following equation [45]:

*b*_nanozyme_ = *V* × (Δ*A*/Δ*t*)/(*ε* × *l*) (1)

where *V* is the volume of reaction mixture (μL), *ε* is the molar absorption coefficient of the colorimetric substrate, *ε_oxidized_*_ABTS,420nm_ = 36,000/(M⋅cm), *l* is the pathlength of light in the cuvette (cm), Δ*A*/Δ*t* (absorbance units min^−1^) is the initial change rate in the absorption at 420 nm, as determined from the *A*-*t* experimental curves, and *b*_nanozyme_ is the catalytic activity expressed in units, where one unit represents the amount of nanoparticles that can catalyze the reaction producing 1 μmol of product per min at 37 °C. The background reaction data (without Mn-PB or PB NCPs in the reaction mixture) were subtracted in the calculation. The specific activity (SA) of the nanoparticles was then calculated using the following equation [45]:

*a*_nanozyme_ = *b*_nanozyme_/*m* (2)

where *m* is the mass (mg) of nanoparticles in each assay.

#### 2.4.2. Peroxidase Kinetics Study

The procedure for the kinetics study was similar to that described in Section 2.4.1 but with a fixed concentration of the Mn-PB or PB NCPs and various concentrations of ABTS substrate in cuvettes. Amounts of 50 μL 125 μg mL^−1^ Mn-PB or PB NCPs in a pH 2.0 buffer and 150 μL 30 wt% H_2_O_2_ were added to sample cuvettes. Different volumes (15–180 μL) of the 10 mg mL^−1^ ABTS solution were used for the kinetics study. The final volume of the reaction mixture in the cuvette was 1250 μL. For the reference cuvette, 150 μL DI water was added instead of 30 wt% H_2_O_2_. The catalytic reaction of ABTS oxidation was recorded by detecting the absorption intensity at 420 nm on a UV-Vis spectrophotometer. By using different concentrations of ABTS in the reaction mixture, a series of *A*-*t* curves was obtained. The initial change rates in the absorption at 420 nm, Δ*A*/Δ*t*, as determined from the *A*-*t* experimental curves for each ABTS concentration, were plotted against the ABTS concentration and fitted with the Michaelis–Menten equation. Additionally, the linear double-reciprocal plots (the Lineweaver–Burk plots) of the Michaelis–Menten curves were built to obtain more exact values of *V_max_* and *K_m_*.

#### 2.4.3. Peroxidase Kinetics Study with Horseradish Peroxidase

Amounts of 25 μL 250 μg mL^−1^ HRP in a pH 6.8 phosphate buffer and 50 μL 2.9 mM H_2_O_2_ were added to the sample cuvette with 1.45 mL of ABTS in a pH 5 phosphate buffer. Different concentrations of ABTS were used: 0.095, 0.381, 0.667, 1.238, 1.905, 2.857, 4.286, 5.714, and 7.619 mM. The final volume of the reaction mixture in the cuvette was 1.525 mL. For the reference cuvette, 25 μL of a pH 6.8 phosphate buffer was added instead of the enzyme solution. The Michaelis–Menten dependence and its linear double-reciprocal graph were built as described in Section 2.4.2, and *V_max_* and *K_m_* were determined from the linear double-reciprocal graph.

#### 2.4.4. Oxidase-like Activity Assay

Amounts of 950 μL pH 2.0 buffer, 250 μL 10 mg mL^−1^ ABTS substrate in buffer, and 50 μL 125 µg mL^−1^ Mn-PB or PB NCPs in buffer were added to the 0.5 cm quartz cuvettes to perform the measurements. The reference cuvette contained 1000 μL pH 2.0 buffer and 250 μL 10 mg mL^−1^ ABTS substrate in buffer. All solutions were incubated at 37 °C in a water bath. The reaction was followed by detecting the absorption intensity at 420 nm on a UV-Vis spectrophotometer. 

#### 2.4.5. Catalase Assay

The catalase-like activities of the Mn-PB or PB NCPs were studied by detecting the O_2_ concentration with an oxygen sensor. The reaction mixture with a total volume of 10 mL contained 1.2 mL 30 wt% H_2_O_2_ and 0.1 M pH 7.4 buffer. The oxygen sensor was immersed in the reaction mixture to detect the dissolved O_2_ concentration change for different concentrations of Mn-PB or PB NCPs.

#### 2.4.6. Superoxide Radical Anion Scavenging Activity 

The O_2_^●−^ scavenging activities of the NCPs were studied using the cytochrome *c* assay, where one-electron oxidation of xanthine catalyzed by xanthine oxidase [14,46] was used to produce a superoxide radical anion. The rate of the reduction of ferricytochrome *c* to ferrocytochrome *c* was followed at 550 nm. In this assay, 0.0655 mM xanthine and 0.005 units xanthine oxidase in 0.05 M pH 7.4 buffer were used to generate O_2_^●−^, while 20 μg mL^−1^ catalase was used to exclude the influence of simultaneously generated H_2_O_2_. The rates of reduction of 0.013 mM cytochrome *c* with different NCP concentrations were followed at 550 nm in the reaction mixture. In additional experiments, 1 unit mL^−1^ SOD enzyme or 5 µg mL^−1^ PVP were tested instead of NCPs. An amount of 0.13 mM ethylenediaminetetraacetic acid disodium salt dihydrate (EDTA⋅2Na) was also added to the reaction mixture to exclude any influence due to possible sources of free iron ions in the solution other than the NCPs.

### 2.5. Preparation of the Glassy Carbon Electrode (GCE)

The GCE was washed with ethanol/water and water under ultrasonication. The surface was then polished using 1 μm and 0.05 μm polishing powder, respectively, before it was thoroughly rinsed with water and dried. The Mn-PB or PB NCPs were dispersed in pH 3.0 HCl solution and dropped onto the surface of the GCE. The electrode with NCPs was subsequently dried in the air for 90 min before 0.25 wt% Nafion solution coating was applied. Finally, the obtained GCE was dried overnight.

## 3. Results

**Figure 1:**
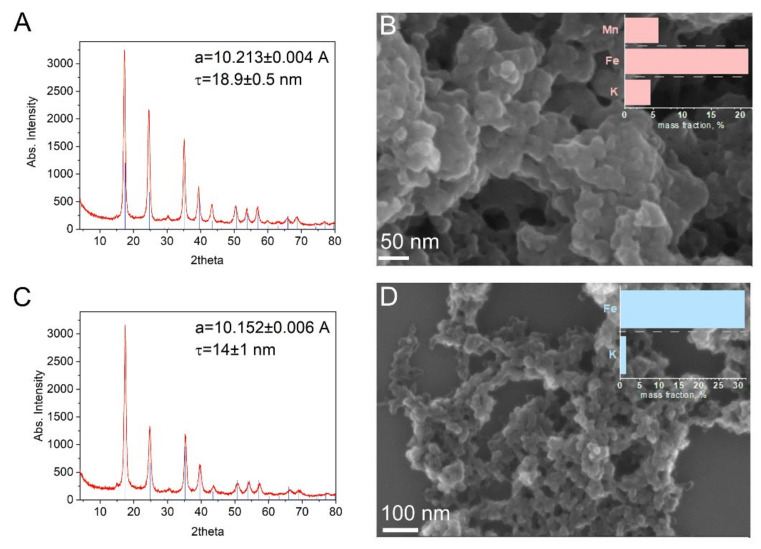
XRD analysis of (**A**) Mn-PB NCPs and (**C**) PB-NCPs, a_reference material_ = 10.199 Å. SEM images of (**B**) Mn-PB NCPs and (**D**) PB NCPs with the corresponding mass fraction diagrams.

**Figure 2:**
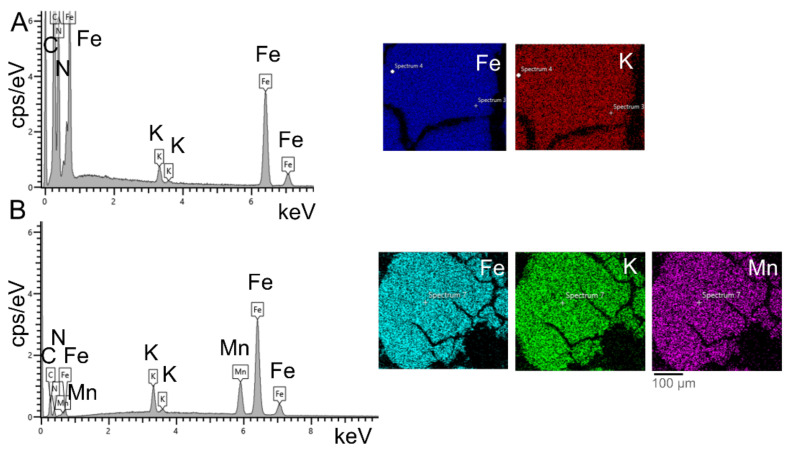
EDX element mapping of (**A**) PB NCPs and (**B**) Mn-PB NCPs.

The nanoparticles were synthesized by means of the reduction, self-assembly, and precipitation of molecular precursors, as described in Section 2.2, without using a stabilizing agent to determine their intrinsic activities. The structure and surface morphology of the Mn-PB and PB NCPs were analyzed by means of XRD and SEM (Figure 1). The XRD analysis (Figure 1A,C) revealed that the materials were well crystallized. The diffraction peak patterns were assigned to the face-centered cubic structure (Fm-3m) with slightly larger dimensions of 10.213 Å for the unit cell and a crystallite size of 18.9 nm for the Mn-PB NCPs in comparison with 10.152 Å and 14 nm for the PB NCPs, respectively (Table S1). Due to the absence of a surface capping agent, the NCPs appeared agglomerated in SEM and had an average particle size below 100 nm (Figure 1B,D). Element mapping from the EDX analysis showed a homogeneous atomic distribution of all metal ions over the PB NCP and Mn-PB NCP samples (Figure 2), while XPS (Figure S1) and ICP-OES analyses (Figure 1B,D and Table S2) revealed a more detailed chemical composition with a weight ratio of K:Mn:Fe 4.37:5.8:21.2 wt% and K:Fe 1.45:31.57 wt% for the Mn-PB and PB NCPs, respectively.

The UV-Vis absorbance spectra in pH 2 and 7.4 buffers are shown in Figure S2A,B,D,E. An absorbance shoulder located at 420 nm and a broad absorption band can be observed in a wavelength range of 700–800 nm with a maximum at about 750 nm for the PB NCPs for both pHs. This maximum shifted slightly bathochromically to 770 nm for the Mn-PB NCPs. The stability tests of the non-stabilized Mn-PB and PB NCPs in pH 2 and 7.4 buffer solutions (Figure S2C,F) revealed some differences between the two types of NCP. In the acidic solution, both types of NCPs had a higher stability than at pH 7.4. After 10 days in the pH 2 buffer, both types of NCP still possessed about 100% initial absorbance, while both PB and Mn-PB NCPs showed poor stability in the pH 7.4 buffer, where only 57.8% (PB) and 40% (Mn-PB) of initial absorbances remained after 10 days.

The peroxidase-like activity of the non-stabilized NCPs was evaluated using a common peroxidase donor substrate ABTS in optimum condition [47,48], as described in Sections 2.4.1 and 2.4.2. Both Mn-PB NCPs and PB NCPs achieved biomimetic oxidation of ABTS with hydrogen peroxide to produce an oxidation product ABTS^●+^ with an absorbance maximum in the region of 420 nm, according to the following reaction [48]:

(3) $$H_{2}O_{2}\;+\;2\mathrm{ABTS}\;\overset{TMC,\;2H^{+}}{\to }\;H_{2}O+{\mathrm{ABTS}}^{\text{ ● }+}\;$$

**Figure 3:**
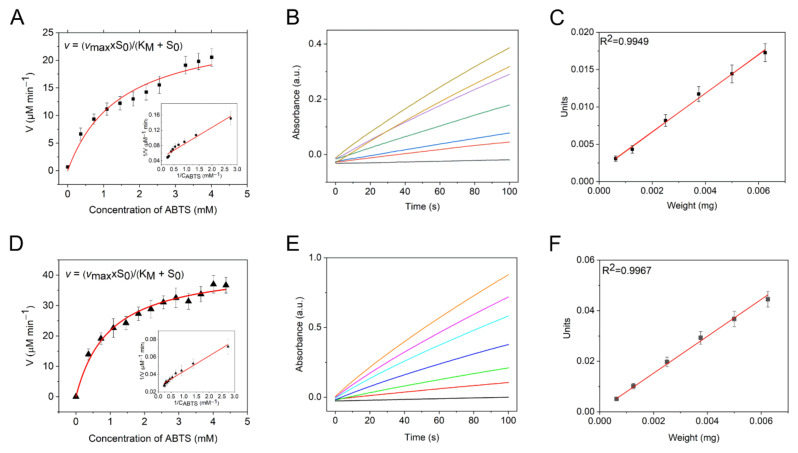
Peroxidase-like activity of the non-stabilized (**A**–**C**) Mn-PB and (**D**,**E**) PB NCPs: (**A**,**D**) kinetics of the ABTS substrate oxidation, 5 µg mL^−1^ NCPs, inserts in (**A**,**D**) show the corresponding Lineweaver–Burk plots, (**B**,**E**) monitoring the ABTS oxidation by the NCPs-H_2_O_2_ system at 420 nm, initial range of the change in the absorbance for 0, 0.5, 1, 2, 3, 4, and 5 µg mL^−1^ NCPs (black line: 0 µg mL^−1^), and (**D**,**F**) specific activity of the NCPs at 3.6 mM ABTS.

The reaction was monitored by following the absorbance intensity at 420 nm. The absorbance increased slowly without the addition of nanoparticles, indicating a moderate reaction between H_2_O_2_ and ABTS. When PB or Mn-PB NCPs were added to the reaction mixture, the change rate of absorbance increased, indicating an accelerated reaction rate between ABTS and H_2_O_2_ due to the addition of NCPs. The nanozymes’ peroxidase-like reactions were found to obey Michaelis–Menten kinetics, which is used to characterize the enzymatic rate at different substrate concentrations (Figure 3A,D). The parameters of the Michaelis–Menten equation, where *V_max_* is the maximal reaction rate of the enzymatic reaction at the saturating substrate concentration, and *K_M_* is the Michaelis constant, were obtained using the linear double-reciprocal plots (the Lineweaver–Burk plots) in Figure 3A,D. To further evaluate the specific catalytic activity, the peroxidase-like activity was calculated as described in Section 2.4.1 and Figure 3B,C,E,F. It can be seen in Figure 3C,E that for both types of NCP, the peroxidase-like activity expressed in units has a linear relationship with the NCP amount. However, the slope of the samples containing PB NCPs is higher than that of the samples containing Mn-PB NCPs, indicating a higher peroxidase-like activity for the first type. Based on this higher reaction rate, the PB NCPs had an SA value of 7.2 units mg^−1^, while the Mn-PB NCPs had an SA value of only 2.6 units mg^−1^. The value of *K_M_* was also lower for the Mn-PB NCPs. The results are shown in Table 1.

To draw further conclusions about the mechanism of the catalyzed reaction, the oxidase activity of the NCPs in oxidizing the ABTS substrate using molecular oxygen as an electron acceptor was examined (Figure S4). It is clear that the Mn-PB and PB NCPs did not possess an oxidase-like activity in this case. Therefore, the catalysis of H_2_O_2_ oxidation in the experiments above is due to the peroxidase-like activity of the NCPs.

As summarized in a number of reviews [32,49,50], the mechanism of the peroxidase-like activity is based on the Fenton reaction, which initiates free radical formation and a redox cycle between different valence states of iron:

≡Fe(II) + H_2_O_2_ → ≡Fe(III) _BG,PY_ + OH^−^ + HO^● ^ (4)

≡Fe(III) + H_2_O_2_ → ≡Fe(II) + H^+^ + HOO^● ^ (5)

2 H_2_O_2_ → H_2_O + HO^● ^ + HOO^● ^ (6)

HO^● ^ + H_2_O_2_ → H_2_O + HO_2_^● ^, pK_a_ (O_2_^●−^) = 4.7 (7)

**Figure 4:**
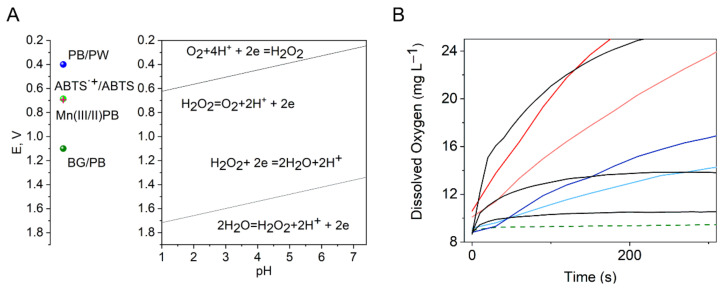
(**A**) E-pH diagram, the data for hydrogen peroxide were obtained from [56]. (**B**) Oxygen production over time catalyzed by the non-stabilized Mn-PB NCPs (red lines), PB NCPs (blue lines)—with the light lines representing 2.5 µg mL^−1^ and the dark lines representing 5 µg mL^−1^ NCP concentration—and catalase (black lines) in concentrations of 0.025, 0.1, and 0.25 µg mL^−1^; dashed line corresponds to the measurement without addition of the NCPs.

≡Fe(III) + HO_2_^● ^ → ≡Fe(II) + O_2_ + H^+^ (8)

where step (5) in the case of Fe(III) was expected to have a lower reaction rate constant than step (4) (Fenton reaction) [49,51,52]. Step (4) resulted in the formation of BG/PY modifications. As shown below, E_1/2_(PB/PW NC) was about 0.4 V (SHE), with no significant pH dependence in the studied pH range, and Mn(III/II) was about 0.690 V. Therefore, the PB and Mn-PB NCs did not directly oxidize ABTS, which has a relatively high redox potential E_1/2_(ABTS^●+^/ABTS) of 0.68–0.69 V (SHE) [53,54], while BG/PB has more positive E_1/2_ values of about 1.1 V (SHE), being a strong oxidizing agent that can oxidize ABTS. This is consistent with the lack of oxidase-like activity of the NCPs toward the ABTS substrate, as shown in Figure S4. However, the oxidizing and reducing properties of H_2_O_2_ significantly depend on the pH being −0.0591pH (Figure 4A). At pH 2, E^0^(H_2_O_2_/H_2_O) is about 1.66 V, and hydrogen peroxide can act as an oxidizing agent at more negative potentials, while E^0^(O_2_/H_2_O_2_) is about 0.56 V, and above this potential, it can act as a reducing substance with an area of double instability (Figure 4A). According to our experiments, ABTS was slowly oxidized by H_2_O_2_ at pH 2 (background line at 0 µg mL^−1^ NCPs). The oxidation rate was largely accelerated in the presence of NCPs (Figure 3B,E). Therefore, the generated hydroxyl radicals and an oxidized-form BG produced in reactions (4)–(8) can obtain a reduction equivalent from the organic ABTS substrate oxidizing ABTS [27,43,49]. Previous studies found a high value for the reaction rate constant, k = 1.2 × 10^10^ M^−1^ s^−1^, for the reaction HO^● ^ + ABTS → ABTS^●+^ + OH^−^ [43]. Accordingly, the lower peroxidase activity of the Mn-PB NCPs can be explained by the fact that hydrogen peroxide oxidizes Mn(II) in Mn-PB, which reduces the contribution of reaction (4), i.e., formation of the BG, while oxidized Mn has a lower oxidizing power than BG (Figure 4A and Figure 6A). The peroxidase activity of PB NCPs can thus be regulated by doping with a suitable transition metal.

Moreover, non-stabilized NCPs can extend the peroxidase-catalyzed oxidations and assays to the low pH range, where natural enzymes are not functional [47]. On the one hand, this property of the NCPs can be favorably used in practical applications, e.g., wastewater treatment [3], while on the other hand, the peroxidase-like activity of iron oxide nanoparticles based on the formation of the hydroxyl radical in the acidic environment of lysosomes of about pH 4 was shown to be responsible for their cytotoxicity [10]. It was found that the pH drop in some areas close to the respiratory complex in the mitochondrial membrane amounted to up to 3 pH units (acidification) in comparison with the bulk compartment [55]. Accordingly, the Fenton activity and, therefore, cytotoxicity of the PB nanomaterials should be re-examined for biomedical investigations [10,32,55].

No catalase-like activity of the NCPs in the presence of hydrogen peroxide was observed at pH 2. We propose that the reason for this difference in activity is the pH dependence of the redox potentials of hydrogen peroxide. The redox potentials of the Mn-PB and PB NCPs did not demonstrate essential pH dependence, as shown below, and the redox potential of Mn(III/II) in Mn-PB NCPs fell into an area of double instability of hydrogen peroxide. However, the oxidizing power of the hydrogen peroxide decreased with increasing pH (Figure 4A) [56]. At pH 7.4, E^0^(H_2_O_2_/H_2_O) is about 1.34 V, while E^0^(O_2_/H_2_O_2_) is about 0.24 V, with an area of double instability and the decomposition of hydrogen peroxide into water and oxygen. This process of H_2_O_2_ decomposition was significantly catalyzed in the presence of the non-stabilized Mn-PB and PB NCPs, which was evidenced by the increasing concentration of dissolved oxygen in the reaction mixture, as illustrated in Figure 4B, and by the visible formation of oxygen bubbles. It is interesting to note that the dynamics of the dissolved oxygen concentration in the H_2_O_2_ decomposition processes catalyzed by NCPs or catalase were different (Figure 4B). In the case of catalase, equilibrium was reached faster. This may be explained by the different activities and the fact that catalase-catalyzed decomposition is a homogeneous process, while NCP-catalyzed decomposition is a heterogeneous process. In addition, at pH 7.4, the dissolution of the NCPs with surface renewal can proceed simultaneously with the catalytic reaction. The observed catalase activity of the Mn-PB and PB NCPs is presumably based on reactions (9)–(11) and was recorded by monitoring overall O_2_ production in reactions (9)–(11) using an oxygen sensor. Catalase activity of the NCPs, k_cat_, was determined from the slope of the dependence of the observed initial rate constant, k_obs_, of reaction (11) on the concentration of the NCPs, Δk_obs_/Δc(_NCPs_) (Table 1). As follows from Figure 4B and Table 1, in contrast to the peroxidase-like activity, doping the PB NCPs with Mn^2+^ increased the catalase-like activity significantly. We assume that the higher catalase-like activity of the Mn-PB NCPs is due to the catalytic properties of the Mn(II) complexes in hydrogen peroxide and oxygen reduction reactions [14,38,57].

≡M^n+^ + H_2_O_2_ → ≡M^(n+1)+^ + 2OH^−^ (9)

≡M^(n+1)+^ + H_2_O_2_ + 2OH^−^→ ≡M^n+^ + O_2_ + 2H_2_O (10)

2H_2_O_2_ → 2H_2_O + O_2_ (11)

The appearance of catalase-like activity at higher pH values is due to the fact that H_2_O_2_ is a weaker oxidant than at acidic pH, as discussed above, and can be more easily oxidized to oxygen, which means that reaction (10) can be facilitated. The pH dependence of the dual enzyme activity of the non-stabilized NCPs in our study is similar to the properties of the polyvinylpyrrolidon (PVP)-stabilized PB NPs in an earlier work [27]. pH dependence of dual-enzyme mimetic activity of TMC, Fe_3_O_4_, and PS@Au@PB nanomaterials was also observed in other works [10,32,38]. The pH-dependent enzyme mimetic activities of the Mn-PB and PB NCPs can therefore be explained by a combination of the redox and catalytic properties of the metal-doped PB NCPs and the dependence of the redox properties of hydrogen peroxide on pH. 

Finally, the O_2_^●−^ scavenging activity of the non-stabilized NCPs was also evaluated using the cyt *c* reduction assay described in Section 2.4.6. In this assay, xanthine and xanthine oxidase were used as a source of superoxide radical anions according to the following reaction [58]:

(12) $$\mathrm{Xanthine}+O_{2}+H_{2}O\;\overset{XO}{\to }\;\mathrm{Uric}\;\mathrm{acid}+{O_{2}}^{•-}+H^{+}$$

Ferricytochrome *c* was used as a colorimetric substrate. Ferricytochrome *c* reacts with superoxide radicals as follows:

Cytochrome *c*^3+^ + O_2_^•−^ → Cytochrome *c*^2+^ + O_2_ (13)

and the reduction in cytochrome *c*^3+^ is accompanied by an increase in its absorption at 550 nm [59]. Monitoring the reduction in cytochrome *c* at 550 nm allows the SOD-like activity of the PB and Mn-PB NCPs to be evaluated because if the nanoparticles possess SOD-like activity, any produced superoxide radicals—which are substrates of SOD—can undergo an accelerated dismutation reaction as follows:

(14) $${2O_{2}}^{•-}+H^{+}\;\overset{SOD\;\left(PB\;or\;Mn-PB\;NCPs\right)}{\to }\;O_{2}+H_{2}O_{2}\;$$

**Figure 5:**
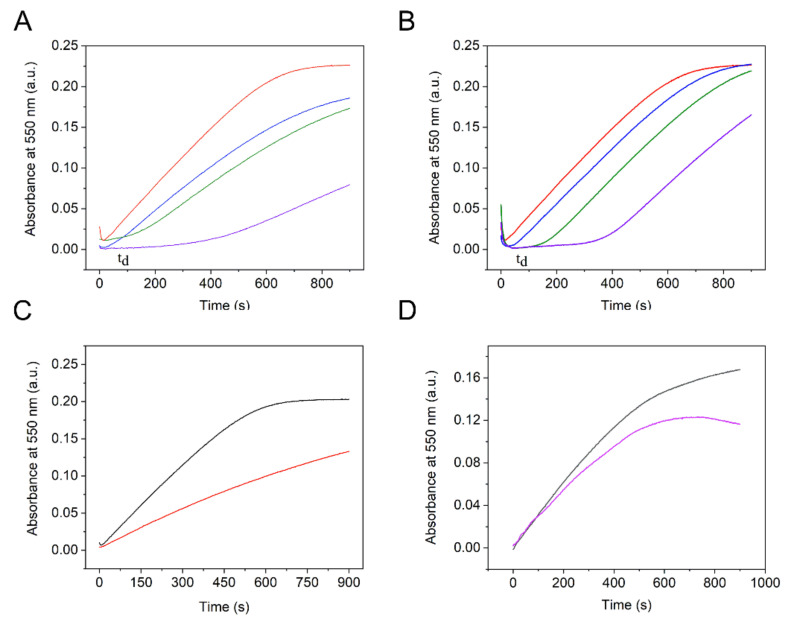
Inhibitive effect of the (**A**) Mn-PB NCPs, (**B**) PB NCPs, (**C**) enzyme superoxide dismutase, and (**D**) PVP on the reduction in ferricytochrome *c* with xanthine–xanthine–oxidase system. In (**A**,**B**): 0 (red lines), 10 (blue lines), 25 (green lines), and 50 (violet lines) µg mL^−1^ of NCPs, (**C**): 0 (black line) and 1 (red line) units mL^−1^ SOD, and (**D**): 0 (black line) and 5 (violet line) µg mL^−1^ of PVP.

O_2_^•−^ can react with cytochrome *c* or disproportionate. A disproportionation reaction will hinder the reduction in cytochrome *c* by competing with it, which means that a decreased change rate in absorption intensity at 550 nm will be recorded in the presence of SOD or NCP catalysts in comparison with the change rate without SOD or NCPs (Figure 5A–C).

As can be seen in Figure 5A,B, without the nanoparticles, the absorption intensity at 550 nm increased steadily within 600 s (red lines), which reflected a stable reaction between cytochrome *c*^3+^ and O_2_^•−^. The curve then became gradually flat after 600 s, indicating an equilibrium. However, with the addition of the NCPs, the reduction process of cytochrome *c* was delayed. As displayed in Figure 5B, the absorption intensity started to increase after 40 s for the sample with 10 µg mL^−1^ PB NCPs. For the samples with 25 and 50 µg mL^−1^ PB NCPs, the reaction was delayed for 130 s and 320 s, respectively. After different periods of delay, the absorption intensities of three samples containing different concentrations of NCPs increased. Based on the delay of the absorption change and the relationship between concentration and delay time, it is possible that O_2_^•−^ reacted with the NCPs, thus hindering the reduction in cytochrome *c*^3+^ and the change in absorption intensity. Earlier experiments showed that PB does not oxidize cyt *c* [27]. Processes (15)–(18) described below may explain the O_2_^●−^ scavenging activity of the NCPs [14,27]. Fast disproportionation (16) (k = 2 × 10^5^ M^−1^ s^−1^ [60]) led to the formation of hydrogen peroxide, which oxidized the reduced metal forms of NCPs in reaction (17). It was shown that the Mn(II) complexes participate in the reduction in oxygen and hydrogen peroxide, being oxidized to Mn(III). The formed Mn(III) complexes can be reduced by O_2_^●−^ and inhibit the reduction in cyt *c* [12,14,46]. The formation of hydrogen peroxide may additionally inhibit the reduction in cyt *c* and activate the catalase activity (9)–(11) of the NCPs at this pH, which highlights the “multifaceted” properties and multiple mechanisms of the NCPs.

M^3+^ + O_2_^•−^ → M^2+^ + O_2_ (15)

O_2_^•−^ → O_2_ + H_2_O_2_ (16)

M^2+^ + H_2_O_2_ → M^3+^ + OH^−^ (17)

M^x+^ + xOH^−^ → M(OH)_x_ (18)

Unexpectedly, the dynamics of the cyt *c* reduction after the introduction of the NCPs differed significantly from those observed for other transition metal complexes [14,46] or superoxide dismutase enzyme (Figure 5B) in similar experiments. The essential difference was manifested in a time delay, t_d_, after which the reduction in cyt *c* started. The reduction rates after the time delay remained lower than those in the absence of NCPs. The time delay in the reduction process followed by different changes in the velocity of cyt *c* reduction at higher NCP concentrations can be explained as follows. Initially, there were many free Fe^III^-sites on the surface of the NCPs, which were reduced by the produced O_2_^●−^ consuming it in reaction (15), thereby inhibiting the reduction in cyt *c*. During processes (15)–(18), PW or hydroxide layers formed on the surface of the non-stabilized NCPs at this pH, which inhibited the fast consumption of O_2_^●−^ by the NCPs while the bulk was still in PB form. The superoxide radical anion did not diffuse through the surface hydroxide layer, and the reduction in cyt *c* began. At the same time, the surface hydroxide passed into the solution near-electrode layer, which provided some new Fe^3+^ on the NCPs, with the result that the cyt *c* reduction continued at a low reduction rate. Processes (9)–(11) and (16)–(18) additionally contributed to the kinetics of the cyt *c* reduction, while both M(II) and cyt *c* were oxidized by the produced hydrogen peroxide. Interestingly, the t_d_ values for the PB and Mn-PB NCPs were similar, which is in agreement with the proposed mechanism, while the kinetics of the subsequent cyt *c* reduction differed significantly. The O_2_^●−^ scavenging activity of the NCPs is compared in Table 1 based on time delay, t_d_, and velocity change after the time delay, V_NCP_/V_0_, where V_NCP_/V_0_ were found from a linear portion of the curves in Figure 5. It can be concluded that the Mn-PB NCPs are stronger scavengers of O_2_^●−^ due to the redox properties of manganese ion, e.g., reactions (15) and (17), the difference in hydroxide formation, and the kinetics of reactions (15) and (17)–(18).

It should be noted that this catalytic behavior of the PB NCPs differs from the previously published results using PVP-stabilized cube-like PB nanoparticles [27]. PVP is often used in the synthesis of NPs and medical applications. We examined the influence of PVP on the cyt *c* reduction in the assay.Figure 5D shows that both the reduction velocity of cyt *c* and A_max, 550_ (reduced cyt *c*) decreased in the presence of PVP, which indicates that cyt *c* reduction is influenced by PVP. Since PVP participates in the reaction of free-radical polymerization toward a higher cross-linked PVP polymer, this process can explain the consumption of the superoxide radical anion that would otherwise reduce cyt *c* (III). This finding implies that the SO scavenging activity of the PVP-stabilized NPs may also receive an essential contribution from the stabilizing agent.

**Figure 6:**
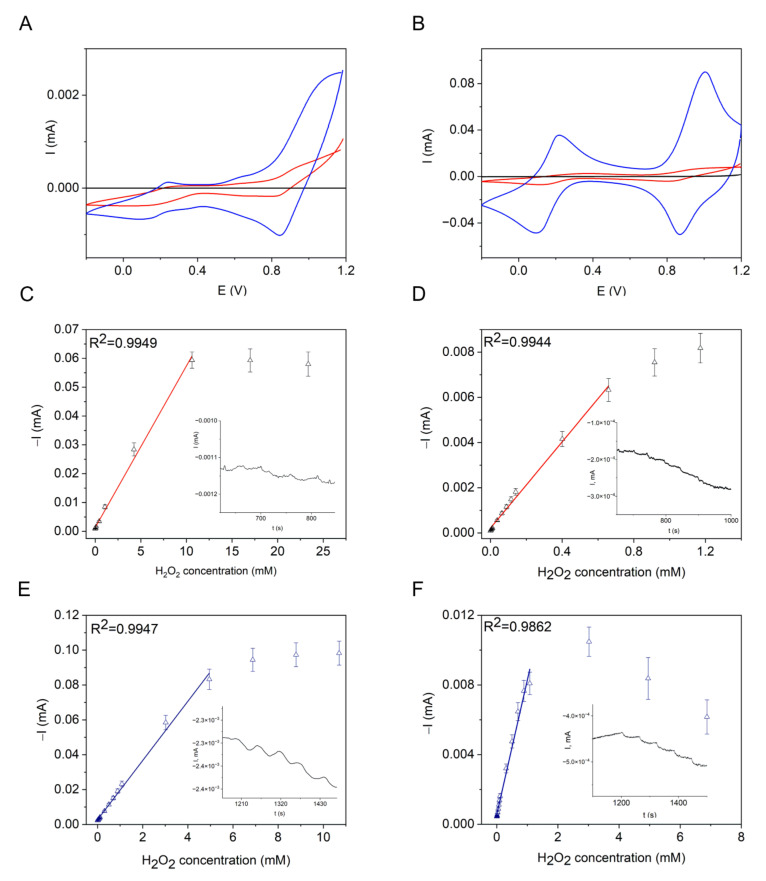
CVs of (**A**) GCE/Mn-PB NCPs/Nafion, (**B**) GCE/PB NCPs/Nafion in the deoxygenated solutions, at pH 3 (blue lines) and pH 7.4 (red lines), 25 mV s^−1^, unmodified GCE at pH 3 (black lines). (**C**–**F**) Calibration curves of the electrodes modified with NCPs: (**C**,**D**) GCE/Mn-PB NCPs/Nafion, −0.05 V, and (**E**,**F**) GCE/PB NCPs/Nafion, 0 V, at pH 3 (**C**,**E**) and pH 7.4 (**D**,**F**), inserts show the amperometric response in the low concentration range.

Finally, the electrochemical properties and electrocatalytic activity of the non-stabilized Mn-PB and PB NCPs toward hydrogen peroxide electroreduction were studied. Electrochemical characterization was performed at pH 3 and 7.4. The first value was chosen because the best stability of PB in electrochemical experiments was previously reported at this pH [22,61], while the second value corresponds to the physiological pH range. Figure 6A,B and Table 2 demonstrate that the electrodes modified with both types of NCPs enable a direct electron transfer similar to the electrodeposited films of PB. Direct electron transfer is often impeded in the case of enzymatic biosensors due to the insulating macromolecular shell of enzymes, and an additional mediator is required.

Figure 6C–F show the amperometric responses and calibration curves of the GCEs modified with Mn-PB and PB NCPs to hydrogen peroxide at pH 3 and 7.4. For comparison, the amperometric response of a bare GCE is shown in Figure S5. In the presence of hydrogen peroxide and in the potential region of the NCP reduction (−0.05–0.05 V), the redox cycle is responsible for the increase in the cathodic current:

PB + e^−^ → PW (19)

PW + H_2_O_2_ → PB + OH^−^ + HO^● ^ (20)

while further reactions may include (5)–(8). Recently, PVP-stabilized MnHCF was also tested for the detection of H_2_O_2_ [62]. However, the detection was carried out based on the oxidation of hydrogen peroxide at 0.65 V, which did not exploit the advantage of PB as an electrocatalytic sensor material; to do so, the detection should be based on hydrogen peroxide reduction catalyzed by PB at potential values close to 0 V. Detailed scanning electrochemical microscopy (SECM) investigations by Noël et al. [31] showed that the electrocatalytic reduction in peroxide at the electrochemically deposited PB layer was accompanied by the release of hydroxyl radicals, [Fe(CN)_6_]^4−^, and the formation of Fe(OH)_3_ and Fe(OH)_2_. Accordingly, PB is often reported to have low stability as a sensor material in neutral and alkaline media, which is due to its dissolution and to a local increase in pH with the formation and precipitation of Fe(OH)_3_ and Fe(OH)_2_, resulting in the progressive loss of PB. This is manifested in the current decrease at pH 7.4, for example (Figure 6F). In this way, localized OH^−^ production was used to pattern the PB layers [31]. Moreover, the generated OH^● ^ (reaction (20)) destroyed organic film in the vicinity of the PB layer [31]. A similar process may be responsible for the degradation of Nafion coatings in sensor devices. The PB-based materials therefore demonstrated low operational stability due to the above-mentioned processes, although the results also show their obvious applicability for the construction of single-use devices. Table 3 summarizes a number of electrochemical characteristics of the GCE/PB/Nafion and GCE/Mn-PB/Nafion sensors at pH 3 and pH 7.4 based on hydrogen peroxide reduction, such as lower detection limit, LDL, sensitivity coefficient, S, and working concentration range, WR. The sensors have comparable characteristics; however, the sensitivity coefficient in acidic medium is almost two times higher for the sensor based on the PB NCPs, most likely due to the higher peroxidase activity of the PB NCPs compared to the Mn-PB NCPs. In addition, as summarized in Table 3, the higher stability of the NCPs and stronger oxidizing properties of hydrogen peroxide in acidic medium are reflected in the higher sensitivity, lower detection limit, and wider working concentration range obtained at pH 3 than 7.4. The LDL_H2O2_ of the NCPs is comparable with that of the biosensors based on various plant peroxidases [34,63]. It can be concluded that the electrocatalysis of hydrogen peroxide reduction by Mn-PB and PB NCPs is reminiscent of their peroxidase-like catalytic substrate oxidation and the cytotoxicity of iron oxide in terms of the destructive action of the produced hydroxyl radicals.

Table S3 compares properties of the NCPs and other Fe-based nanozymes as reported in the literature. The table displays a great diversity in the observed data. The PB NCPs investigated in this work have values of *K_m_* and *V_max_* comparable with those for some magnetite and PB nanozymes. However, many nanozymes investigated in other works contain stabilizing agents on their surface, which can contribute to the observed properties, and there is a large structural diversity in the nanozymes. These variations make it difficult to determine the influence of a particular parameter on the properties of nanozymes. Moreover, specific intrinsic activities, which is an important characteristic for analytical applications, and electrochemical properties have not been reported in most of these works. In many studies, only peroxidase-like activity was investigated. As we showed in this work, changes in the background, such as pH and substrate, lead to the manifestation of different enzyme-like properties due to the combination of the redox properties of the reagents, with the mechanisms becoming complicated because of a number of parallel processes. Furthermore, using a non-stabilized design of NPs with similar structure, we showed that doping the PB mixed-valence coordination compound with other transition metals may represent an approach to tune its intrinsic multienzyme activities and use the reactions mediated by reactive oxygen species in various assays. The properties of nanozymes based on the PB analogs will depend on the stability of the crystal and the redox and catalytic properties of the components, as well as their synergy. Moreover, the relationship between the properties of NPs in the optical and electrochemical assay has not been established so far because most studies use only one detection mode.

## 4. Conclusions

The NCPs of PB and Mn-doped PB were synthesized without using a stabilizing agent, which allowed us to investigate their intrinsic properties. A comparative study of the structural, redox, and catalytic properties of the NCPs was performed. The effect of transition metal doping on the properties of the non-stabilized PB NCPs was shown. Complex mechanisms of the processes responsible for multienzyme-like activities depending on the pH, substrate, and the redox and catalytic properties of the NCPs were proposed. Although cyano-bridged assemblies are traditionally considered an “artificial peroxidase”, this study highlights the intrinsic “multifaceted” properties of non-stabilized Mn-PB and PB NCPs based on the remarkable biomimetic activities of three major antioxidant enzymes. This study shows that the multienzyme mimetic activities of the PB-based nanozymes can be regulated by doping with a suitable transition metal, which offers one approach to control and use the reactions mediated by reactive oxygen species. While Mn-PB NCPs are characterized by a lower peroxidase activity in comparison with PB NCPs, Mn-PB NCPs demonstrate an essentially higher catalase activity and are stronger scavengers of O_2_^●−^. A quantitative evaluation of the intrinsic enzyme-like activities of the non-stabilized NCPs was provided. A combination of the redox and catalytic properties of the metal-doped PB NCPs, dependence of the redox properties of hydrogen peroxide on pH, and properties of the enzyme substrate explain the intrinsic multienzyme-like activities of the Mn-PB and PB NCPs. The non-stabilized NCPs can extend the peroxidase-catalyzed oxidations and assays to the low pH range, while the electrocatalysis of the hydrogen peroxide reduction by the NCPs is reminiscent of their peroxidase-like catalytic substrate oxidation and the cytotoxicity of iron oxide in terms of the destructive action of the produced hydroxyl radicals. The results of this study may further advance the development and application of TMC nanozymes in a variety of fields, such as the development of analytical methods, catalysis, biomedicine, ecology, and technological control.

## Figures and Tables

**Table 1:** Parameters of the enzyme-like activities of the NCPs.

	Peroxidase-like Activity			Catalase Activity	O_2_^●−^ Scavenging Activity	
	a ^1^, U mg^−1^	K_M_^ABTS^, mM	V_max_^ABTS^, µM min^−1^	k_cat_, s^−1^ mL µg^−1^	t_d_, s	V_NCP_/V_0_
Mn-PB	2.6	0.8	21	0.017	300	0.40
PB	7.2	0.68	38	0.006	320	0.83
HRP	-	0.48 ^2^	23 ^2^	-	-	-

^1^ Found for the concentration of ABTS substrate 3.6 mM; ^2^ found for 1.2 × 10^−3^ units mL^−1^ HRP at pH 6.8, see Figure S3 and Section 2.4.3.

**Table 2:** Electrochemical properties of the NCPs.

	PB/PW ^1^				PY, BG/PB ^1^	
	Ea,V	Ec,V	ΔE, V	I_c_/I_a_	E_a_, V	E_c_, V
PB	0.216	0.105	0.111	1	1002	0.870
Mn-PB	0.238 0.330 ^2^	0.116 0.638 ^2^	0.122	1	1072	0.843

^1^ determined at pH 3, ^2^ Mn(III/II).

**Table 3:** Electrochemical sensor properties of the NCPs based on the hydrogen peroxide reduction.

	LDL_H2O2_, µM		S A/(M⋅cm^2^)		WCR, mM	
	pH 3	pH 7.4	3	7.4	3	7.4
PB	1	3	0.23	0.11	1 × 10^−3^–5	3 × 10^−3^–1
Mn-PB	2	3	0.12	0.14	2 × 10^−3^–10	3 × 10^−3^–0.7

## Data Availability

The data presented in this study are available on request from the corresponding author.

## Supplementary Materials

The following supporting information can be downloaded at: https://www.mdpi.com/article/10.3390/nano12122095/s1, Figure S1: XPS spectra of (A) Mn-PB NCPs, survey, C1s1, N1s2, Fe2p3 5, Mn2p3 5, and (B) PB NCPs, survey, C1s1, N1s2, Fe2p3 5; Figure S2: UV-vis absorbance spectra and stability of the 0.15 mg mL^−1^ non-stabilized (A–C) Mn-PB NCPs and (D–F) PB NCPs at pH 2 (A,D) and pH 7.4 (B,E). (C,F) Dependence of the absorption on the storage time at pH 2 (red symbols) and pH 7.4 (black symbols); Figure S3: Kinetics of the ABTS substrate oxidation catalyzed by HRP; Figure S4: Study of the oxidase-like activity of the Mn-PB and PB NCPs; Figure S5: Amperometric response to hydrogen peroxide of a bare GCE at pH 3 (red line) and pH 7.4 (black line) at 0 V; Figure S6: Kinetics of the TMB substrate oxidation, 5 µg mL^−1^ NCPs: (A) Mn-PB and (B) PB NCPs, inserts in (A) and (B) show the corresponding Lineweaver–Burk plots; Table S1: XRD analysis of the Mn-PB NCPs and PB-NCPs; Table S2: Elemental analysis of the Mn-PB NCPs and PB NCPs by ICP-OE, MW is the mass fraction mean, and SD is the standard deviation; Table S3: Peroxidase-like properties of nanozymes with ABTS substrate. References [64,65,66,67,68,69,70] are cited in the Supplementary Materials.
