# Supplementary material for: Intrinsic Multienzyme-like Activities of the Nanoparticles of Mn and Fe Cyano-Bridged Assemblies
Source: Nanomaterials (Basel). 2022 Jun 17;12(12):2095. doi: 10.3390/nano12122095 (PMC9227851; doi:10.3390/nano12122095)
Supplement: Supplementary file 1 [file nanomaterials-12-02095-s001.zip › Highlights.pdf]

Highlights

- intrinsic multienzyme-like activities of the nanoparticles of Mn and Fe cyano-bridged assemblies
- effect of manganese doping on the structural, biomimetic, and electrocatalytic properties
- electrocatalysis of H_2O_2 reduction is reminiscent of the peroxidase-like activity in terms of the $HO^{\bullet}$ formation
- multienzyme-like activities of PB NCPs can be regulated by doping with a suitable transition metal
