# Supplementary material for: Intrinsic Multienzyme-like Activities of the Nanoparticles of Mn and Fe Cyano-Bridged Assemblies
Source: Nanomaterials (Basel). 2022 Jun 17;12(12):2095. doi: 10.3390/nano12122095 (PMC9227851; doi:10.3390/nano12122095)
Supplement: Supplementary file 1 [file nanomaterials-12-02095-s001.zip › nanomaterials-1723591-supplementary.pdf]

nanomaterials

MDPI

*Supporting Information*

# Intrinsic Multienzyme-like Activities of the Nanoparticles of Mn and Fe Cyano-Bridged Assemblies

**Yunong Zhang, David Kudriashov, Liubov Pershina, Andreas Offenhäusser and Yulia Mourzina ***

Institute of Biological Information Processing (IBI-3-Bioelectronics), Forschungszentrum Jülich, 52425 Jülich, Germany; yun.zhang@fz-juelich.de (Y. Z.); d.kudriashov@fz-juelich.de (D.K.); liubovvldpershina@gmail.com (L.P.); a.offenhaeusser@fz-juelich.de (A.O.)

* Correspondence: y.mourzina@fz-juelich.de; Tel.: +49-2461612364

**Table S1.** XRD analysis of the Mn-PB NCPs and PB-NCPs.

| | | |
|---|---|---|
| (A) Mn-PB NCP | Crystal system | cubic |
| | Space group | *Fm-3m* |
| | a | 10.213±0.004 Å |
| | Crystallite size | τ=18.9±0.5 nm |
| (B) PB NCP | Crystal system | cubic |
| | Space group | *Fm-3m* |
| | a | 10.152±0.006 Å |
| | Crystallite size | τ=14±1 nm |
| (C) Reference material PDF #52-1907 | Crystal system | cubic |
| | Space group | *Fm-3m* |
| | a | 10.199 Å |

A

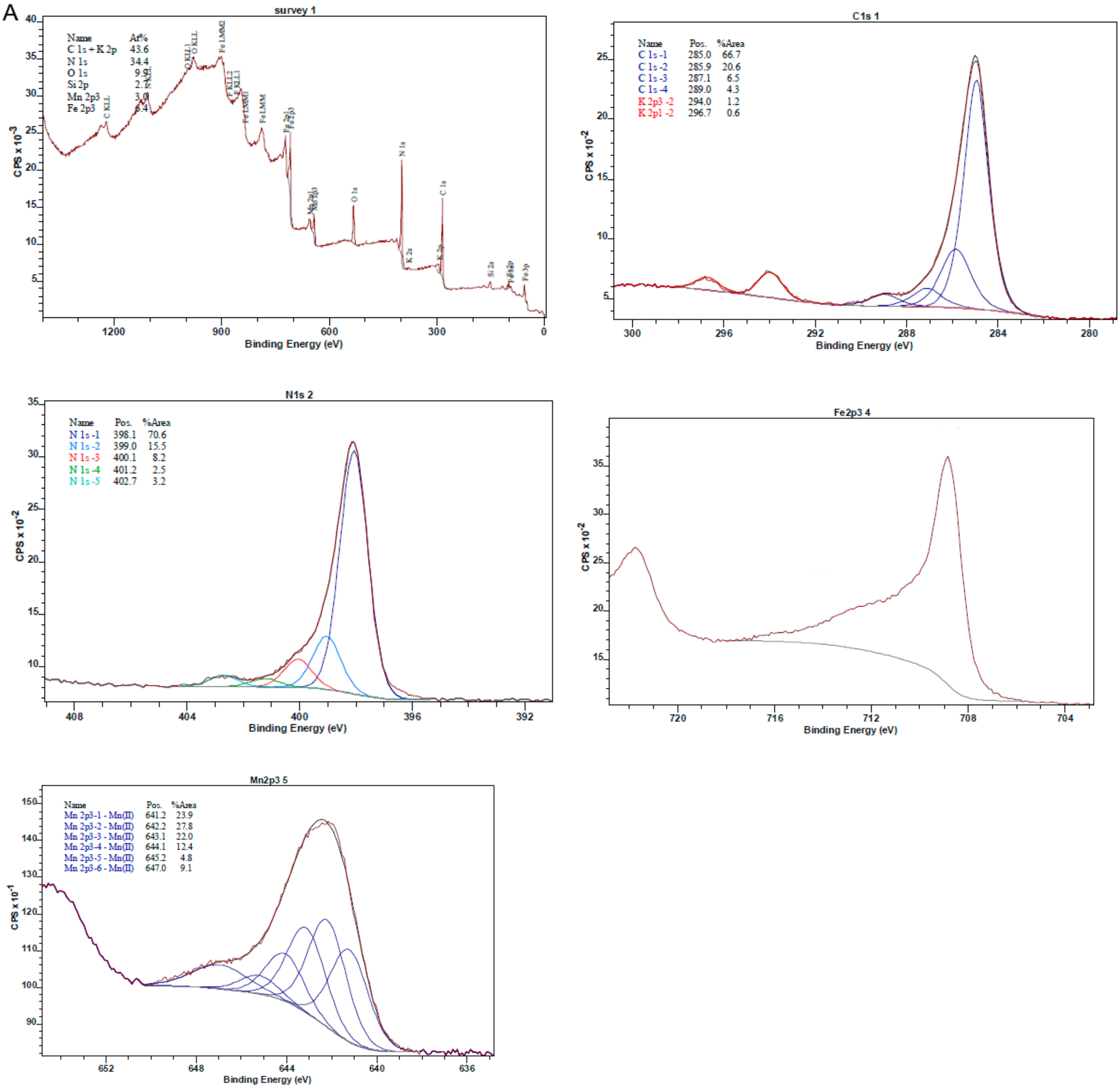
survey 1
Name At%
C 1s + K 2p 43.6
N 1s 34.4
O 1s 9.9
Si 2p 2.7
Mn 2p3 3.0
Fe 2p3 6.4
CPS x 10-3
Binding Energy (eV)
C1s 1
Name Pos. %Area
C 1s -1 285.0 66.7
C 1s -2 285.9 20.6
C 1s -3 287.1 6.5
C 1s -4 289.0 4.3
K 2p3 -2 294.0 1.2
K 2p1 -2 296.7 0.6
CPS x 10-2
Binding Energy (eV)
N1s 2
Name Pos. %Area
N 1s -1 398.1 70.6
N 1s -2 399.0 15.5
N 1s -3 400.1 8.2
N 1s -4 401.2 2.5
N 1s -5 402.7 3.2
CPS x 10-2
Binding Energy (eV)
Fe2p3 4
CPS x 10-2
Binding Energy (eV)
Mn2p3 5
Name Pos. %Area
Mn 2p3-1 - Mn(II) 641.2 23.9
Mn 2p3-2 - Mn(II) 642.2 27.8
Mn 2p3-3 - Mn(II) 643.1 22.0
Mn 2p3-4 - Mn(II) 644.1 12.4
Mn 2p3-5 - Mn(II) 645.2 4.8
Mn 2p3-6 - Mn(II) 647.0 9.1
CPS x 10-1
Binding Energy (eV)

B

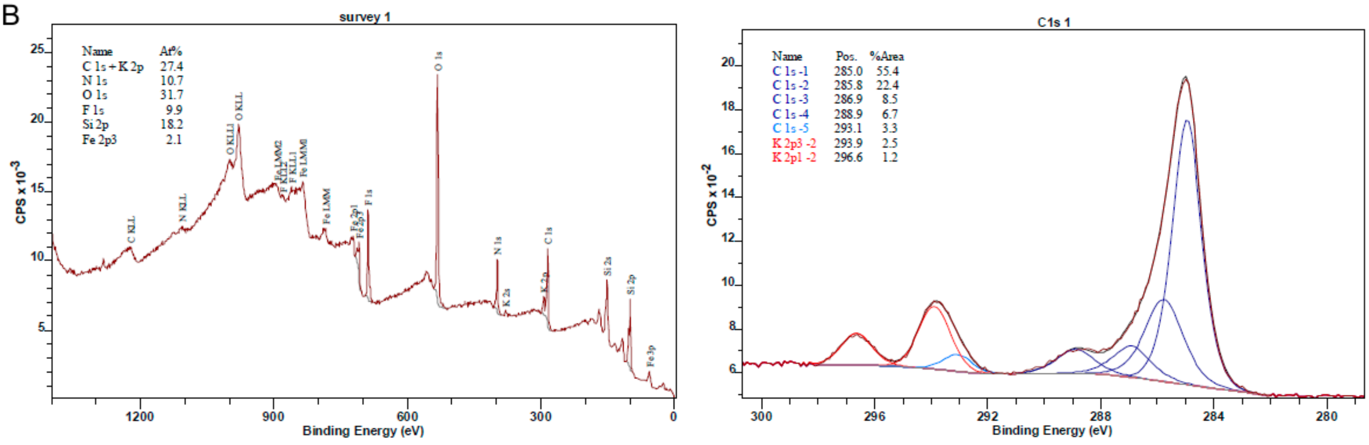
survey 1
Name At%
C 1s + K 2p 27.4
N 1s 10.7
O 1s 31.7
F 1s 9.9
Si 2p 18.2
Fe 2p3 2.1
CPS x 10-3
Binding Energy (eV)
C1s 1
Name Pos. %Area
C 1s -1 285.0 55.4
C 1s -2 285.8 22.4
C 1s -3 286.9 8.5
C 1s -4 288.9 6.7
C 1s -5 293.1 3.3
K 2p3 -2 293.9 2.5
K 2p1 -2 296.6 1.2
CPS x 10-2
Binding Energy (eV)

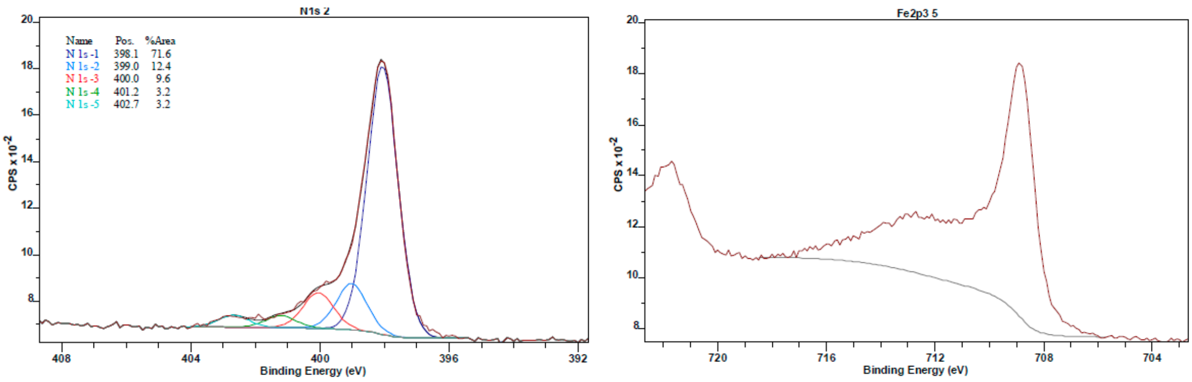


**Figure S1.** XPS spectra of **(A)** Mn-PB NCPs, survey, C1s1, N1s2, Fe2p3 5, Mn2p3 5, and **(B)** PB NCPs, survey, C1s1, N1s2, Fe2p3 5.

**Table S2.** Elemental analysis of the Mn-PB NCPs and PB NCPs by ICP-OE, where MW is the mass fraction mean and SD is the standard deviation.

| PB NCP | | | Mn-PB NCP | | |
|---|---|---|---|---|---|
| | MW, % | SD | | MW, % | SD |
| K | 1,45 | 0,03 | K | 4,37 | 0,19 |
| Fe | 31,57 | 0,08 | Fe | 21,2 | 0,9 |
| | | | Mn | 5,8 | 0,2 |

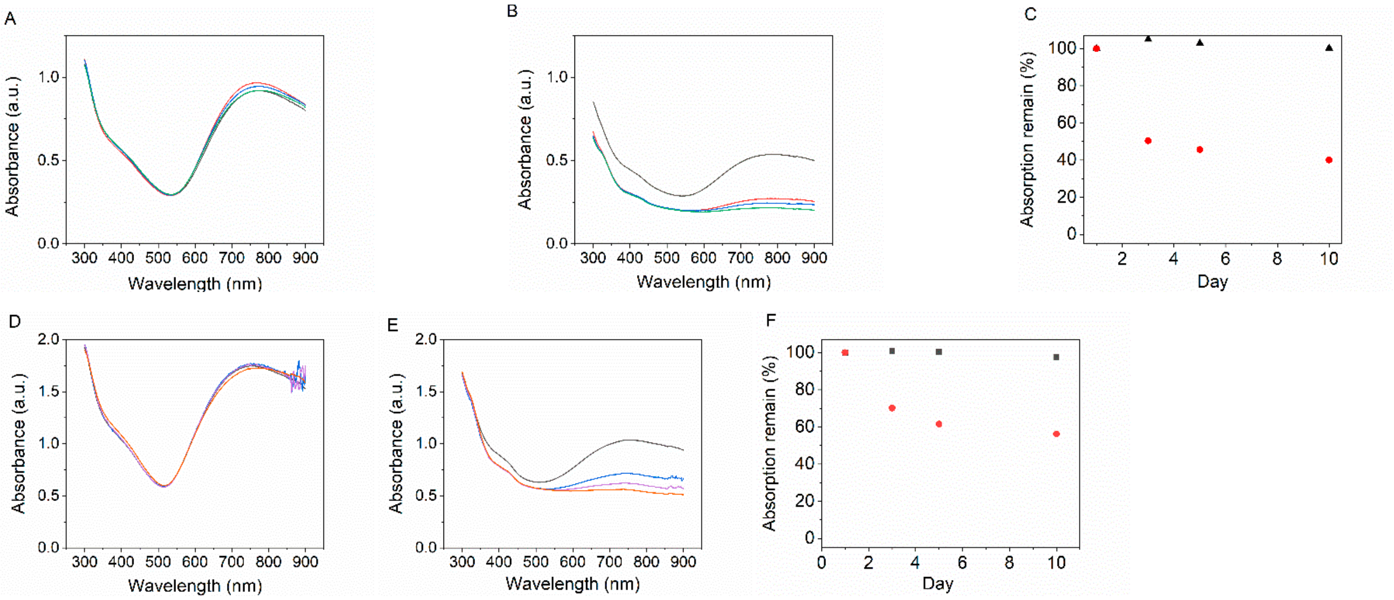


**Figure S2.** UV-Vis absorbance spectra and stability of the 0.15 mg mL$^{-1}$ non-stabilized **(A–C)** Mn-PB NCPs and **(D–F)** PB NCPs at pH 2 **(A,D)** and pH 7.4 **(B,E),** day 1 to 10 – from higher absorbance to lower absorbance. **(C,F)** Dependence of the absorption on the storage time at pH 2 (black symbols) and pH 7.4 (red symbols).

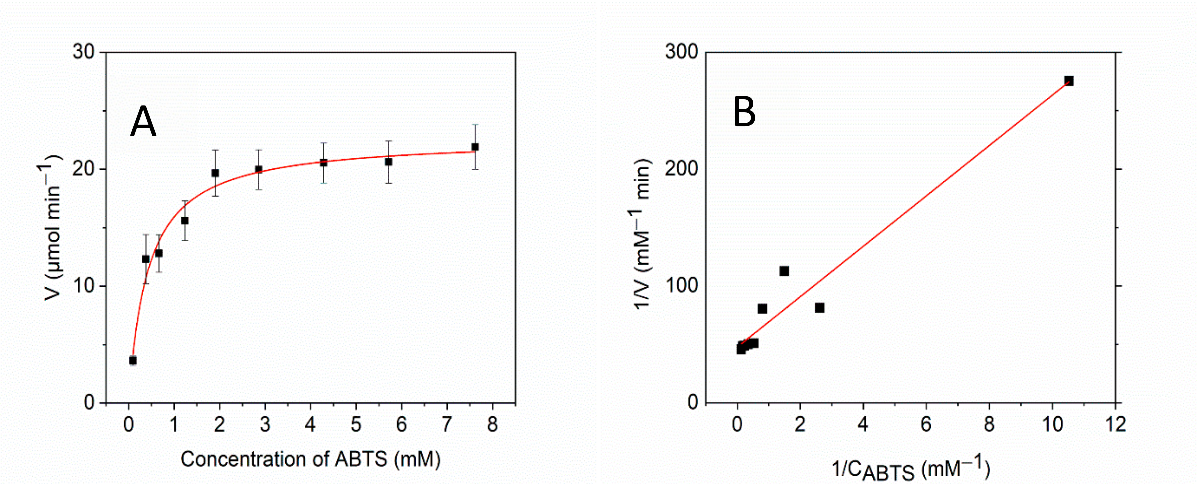


**Figure S3.** Kinetics of the ABTS substrate oxidation catalyzed by HRP (**A**) Michaelis–Menten graph and (**B**) the corresponding Lineweaver–Burk plot. Experimental conditions are described in section 2.4.3.

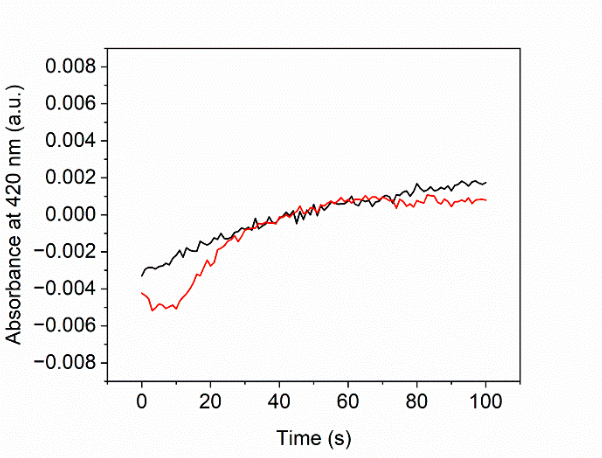


**Figure S4.** Study of the oxidase-like activity of 5 μg $mL^{-1}$ Mn-PB (red line) and PB (black line) NCPs using ABTS as a substrate. Experimental conditions are described in section 2.4.4.

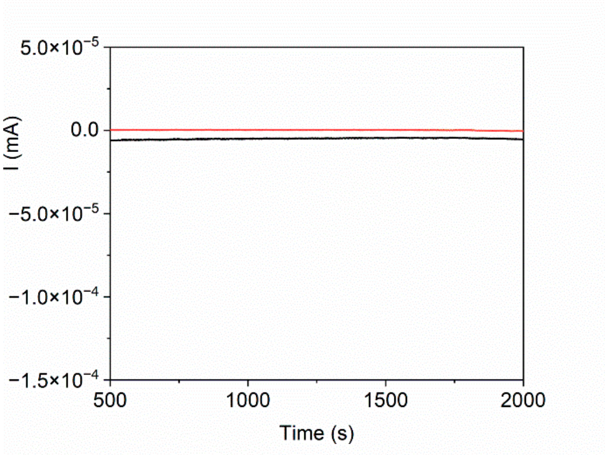


**Figure S5.** Amperometric response to hydrogen peroxide of a bare GCE at pH 3 (red line) and pH 7.4 (black line) at 0 V.

**Table S3.** Peroxidase-like properties of Fe-based-nanozymes.

| Nanozyme | With ABTS as a substrate | | With TMB as a substrate | | S, A/(M·cm²) | Reference |
|---|---|---|---|---|---|---|
| | $V_{max}$, μM min⁻¹ | $K_M$, mM | $V_{max}$, μM min⁻¹ | $K_M$, mM | | |
| PB (PVP) | 18.84 | 1.08 | 13.0 | 0.337 | - | [1] |
| PB | | 157.45 | | | | [2] |
| PB-ferritin | | 0.532 | | | | [3] |
| PB | | | | | 0.85 (pH 6) | [4] |
| PB film | | | | | 0.65 (pH 6) | [4] |
| GQDzyme | 4.53 | 86.96 | | | - | [5] |
| Magnetite Nanoparticles, $Fe_3O_4$ | 263.4 (4.39 μM $s^{-1}$) | 1.22 | | | - | [6] |
| $Fe_3O_4$@Au-Cys-FA | 6.57 (10.95×$10^{-8}$ M $s^{-1}$) | 0.44 | | | - | [7] |
| $Fe_3O_4$* | 10.2 | 0.45 | | | | [8] |
| MNP | | | 10.8 (0.18 μM $s^{-1}$) | 0.142 | | [9] |
| His@MNP | | | 30.6 (0.51 μM $s^{-1}$) | 0.149 | | [9] |
| Fe-rGO sheets | | | 26.8 (0.45 μM $s^{-1}$) | 0.071 | | [10] |
| Fe-N-rGO sheets | | | 104.4 (1.74 μM $s^{-1}$) | 0.074 | | [10] |
| PB | | | 1.62 | 0.91 | | [11] |
| MIL-101(Cr)@PB | | | 6.9 | 0.88 | | [11] |
| PB NCPs | 38 | 0.68 | 99.3 [1] | 5.3 [1] | 0.23 (pH 3) 0.11 (pH 7.4) | this work |
| Mn-PB NCPs | 21 | 0.8 | 101.6 [1] | 12.5 [1] | 0.12 (pH 3) 0.14 (pH 7.4) | this work |

[1] – the data were found as described in section S1 and Figure S6.
GQD – graphene quantum dot, Cys – cysteine, FA – folic acid, $Fe_3O_4$* – acidified colloidal suspension of $Fe_3O_4$; MNP - magnetic nanoparticles; His@MNP – histidine coated magnetic nanoparticles; Fe-rGO – Fe-doped graphene; Fe-N-rGO – Fe–N4 single site embedded graphene.

**Section S1. Peroxidase kinetics study using TMB as a substrate.**

The procedure for the kinetics study was carried out using a fixed concentration of the Mn-PB or PB NCPs and various concentrations of TMB substrate in cuvettes. 50 μL 125 μg $mL^{-1}$ Mn-PB or PB NCPs in a pH 2.0 buffer and 150 μL 30 wt% $H_2O_2$ were added to sample cuvettes. Different volumes (20–300 μL) of the 10 mg $mL^{-1}$ TMB (dissolved in DMSO) solution were used for the kinetics study. The final volume of the reaction mixture in the cuvette was adjusted to 1250 μL using a pH 2.0 phosphate buffer. For the reference cuvette, 150 μL DI water was added instead of 30 wt% $H_2O_2$. The change of absorption was recorded at 652 nm. For calculations, an extinction coefficient of the oxidized TMB of 39000 $M^{-1}$ $cm^{-1}$ was used. By using different concentrations of TMB, a series of *A-t* curves was obtained. The initial change rates of the absorption at 652 nm, ΔA/Δt, as determined from the *A-t* experimental curves for each TMB concentration were plotted against the

TMB concentration and fitted with the Michaelis–Menten equation. Additionally, the linear double-reciprocal plots (the Lineweaver–Burk plots) of the Michaelis–Menten curves were built to obtain more exact values of $V_{max}$ and $K_m$. The experimental results are shown in Figure S6 and the data are summarized in Table S3.

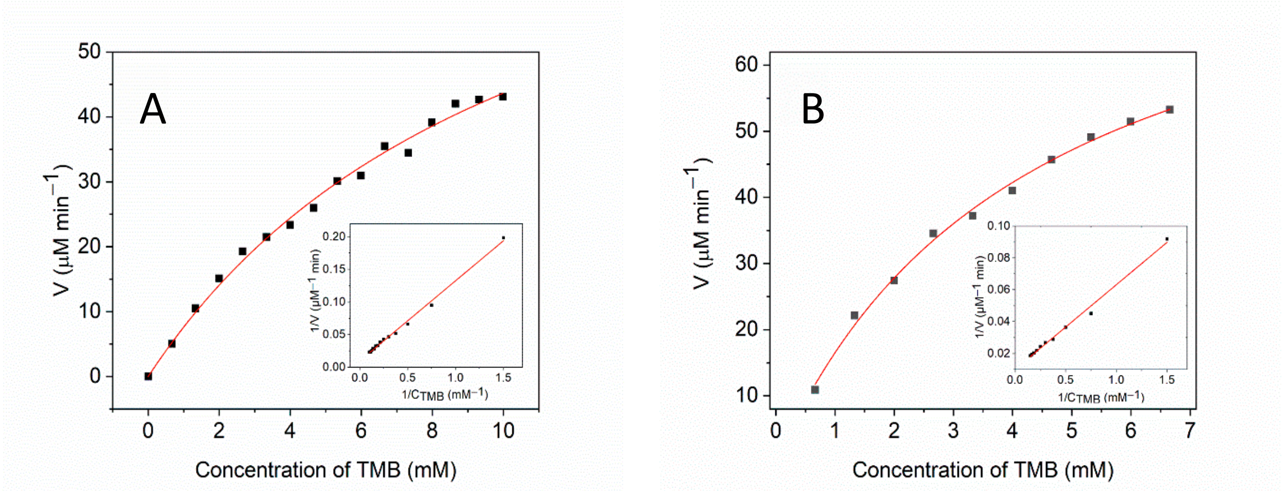


**Figure S6.** Kinetics of the TMB substrate oxidation, 5 µg mL$^{-1}$ NCPs: **(A)** Mn-PB and **(B)** PB NCPs, inserts in **(A)** and **(B)** show the corresponding Lineweaver–Burk plots.

## References

1. Zhang, W.; Hu, S.; Yin, J.-J.; He, W.; Lu, W.; Ma, M.; Gu, N.; Zhang, Y. Prussian Blue nanoparticles as multienzyme mimetics and reactive oxygen species scavengers. *J. Am. Chem. Soc.* **2016**, *138*, 5860–5865.
2. Zhang, W.; Ma, D.; Du, J. Prussian blue nanoparticles as peroxidase mimetics for sensitive colorimetric detection of hydrogen peroxide and glucose. *Talanta* **2014**, *120*, 362–367.
3. Zhang, W.; Zhang, Y.; Chen, Y.; Li, S.; Gu, N.; Hu, S.; Sun, Y.; Chen, X.; Quan, L. Prussian Blue modified ferritin as peroxidase mimetics and its applications in biological detection. *J. Nanosci. Nanotechnol.* **2012**, *12*, 1–8.
4. Komkova, M.A.; Karyakina, E.E.; Karyakin, A.A. Catalytically synthesized prussian blue nanoparticles defeating natural enzyme peroxidase. *J. Am. Chem. Soc.* **2018**. *140*, 11302–11307.
5. Ding, H.; Cai, Y.; Gao, L.; Liang, M.; Miao, B.; Wu, H.; Liu, Y.; Xie, N.; Tang, A.; Fan, K.; Yan, X.; Nie, G. Exosome-like nanozyme vesicles for $H_2O_2$-responsive catalytic photoacoustic imaging of xenograft nasopharyngeal carcinoma. *Nano Lett.* **2019**, *19*, 203–209.
6. Zakharzhevskii, M.; Drozdov, A.S.; Kolchanov, D.S.; Shkodenko, L.; Vinogradov, V.V. Test-System for Bacteria Sensing Based on Peroxidase-Like Activity of Inkjet-Printed Magnetite Nanoparticles. *Nanomaterials* **2020**, *10*, 313.
7. Ponlakhet, K.; Amatatongchai, M.; Sroysee, W.; Jarujamrus, P.; Chairam, S. Development of sensitive and selective glucose colorimetric assay using glucose oxidase immobilized on magnetite–gold–folate nanoparticles. *Anal. Methods* **2016**, *8*, 8288–8298.
8. Liu, Y.; Yu, F. Substrate-specific modifications on magnetic iron oxide nanoparticles as an artificial peroxidase for improving sensitivity in glucose detection. *Nanotechnol.* **2011**, *22*, 145704.
9. Cheon, H.J.; Nguyen, Q.H.; Kim, M.I. Highly sensitive fluorescent detection of acetylcholine based on the enhanced peroxidase-like activity of histidine coated magnetic nanoparticles. *Nanomaterials* **2021**, *11*, 1207.
10. Kim, M.S.; Lee, J.; Kim, H.S.; Cho, A.; Shim, K.H.; Le, T.N.; An, S.S.A.; Han, J.W.; Kim, M.I.; Lee, J. Heme cofactor-resembling Fe–N single site embedded graphene as nanozymes to selectively detect $H_2O_2$ with high sensitivity. *Adv. Funct. Mat.* **2020**, *30*, 1905410.
11. Su, L.; Xiong, Y.; Yang, H.; Zhang, P.; Ye, F. Prussian blue nanoparticles encapsulated inside a metal–organic framework via in situ growth as promising peroxidase mimetics for enzyme inhibitor screening. *J. Mater. Chem. B* **2016**, *4*, 128–134.
